# Ectoine in the Treatment of Irritations and Inflammations of the Eye Surface

**DOI:** 10.1155/2021/8885032

**Published:** 2021-02-09

**Authors:** Andreas Bilstein, Anja Heinrich, Anna Rybachuk, Ralph Mösges

**Affiliations:** ^1^Am Platz 2, 50129 Bergheim, Germany; ^2^bitop AG, Carlo-Schmid-Allee 5, Dortmund, Germany; ^3^Bogomolets National Medical University, Department of Oral and Maxillofacial Surgery, Tarasa Shevchenko Blvd, 13, Kiev, Ukraine 01601; ^4^State Institution “O.S. Kolomiychenko Institute of Otolaryngology of the National Academy of Medical Sciences of Ukraine”, Zoolohichna St, 3, Kiev, Ukraine 03057; ^5^Institute of Medical Statistics and Computational Biology, Faculty of Medicine, University of Cologne, Kerpener Str. 62, 50937 Cologne, Germany; ^6^CRI Ltd., Genter Str. 7, 50672 Cologne, Germany

## Abstract

The ocular surface is facing various unspecific stress factors resulting in irritation and inflammation of the epithelia, causing discomfort to the patients. Ectoine is a bacteria-derived extremolyte with the ability to protect proteins and biological membranes from damage caused by extreme environmental conditions like heat, UV-light, high osmolarity, or dryness. Evidence from preclinical and clinical studies attest its effectiveness in treating several epithelium-associated inflammatory diseases, including the eye surface. In this review, we analysed 16 recent clinical trials investigating ectoine eye drops in patients with allergic conjunctivitis or with other unspecific ocular inflammations caused by e.g. ophthalmic surgery. Findings from these studies were reviewed in context with other published work on ectoine. In summary, patients with irritations and unspecific inflammations of the ocular surface have been treated successfully with ectoine-containing eye drops. In these patients, significant improvement was observed in ocular symptoms of allergic rhinoconjunctivitis, postoperative secondary dry eye syndrome, or ocular reepithelisation after surgery. Using ectoine as an add-on therapy to antihistamines, in allergy patients accelerated symptom relief by days, and its use as an add-on to antibiotics resulted in faster wound closure. Ectoine is a natural substance with an excellent tolerability and safety profile thus representing a helpful alternative for patients with inflammatory irritation of the ocular surface, who wish to avoid local reactions and side effects associated with pharmacological therapies or wish to increase the efficacy of standard treatment regimen.

## 1. Introduction

Ectoine, an extremolyte, is a natural protection molecule found in bacteria which survive under extreme conditions of salinity, drought, irradiation, pH, and temperature [1, 2]. Ectoine forms a protective hydration shield around proteins and other biomolecules [3] that is based on its strong binding capacities to water molecules [4]. This mode of action is known as “preferential exclusion” [5]; i.e., ectoine is preferentially excluded from the hydrate shield, leading to the alteration of the aqueous solvent structure [6, 7]. That effect protects proteins from damage and irreversible denaturation and stabilizes biological membranes [4, 8–10]. In preclinical studies, ectoine was shown to protect lung and skin cells against the damage induced by toxic pollution particles and to prevent the subsequent activation of inflammatory cascades [11–16]. A similar effect was observed in model systems for inflammatory bowel disease [17]. Interestingly, ectoine can stabilize lipid layers in pulmonary surfactants, as well as the tear film of the eyes against physical stress [18–22].

Promising findings from clinical trials were reviewed by Casale and colleagues [23] who attributed topical applied ectoine effectiveness in upper airway inflammations such as acute pharyngitis/laryngitis [24, 25], rhinosinusitis, rhinitis sicca, and acute bronchitis [26]. In addition, several trials showed efficacy of ectoine in various diseases with barrier dysfunctions such as rhinitis sicca [27], chemotherapy-induced mucositis [28], lung inflammation caused by environmental pollutants [29], prevention of upper respiratory infections [30], and atopic dermatitis [31]. Moreover, studies on allergic rhinoconjunctivitis [27, 32, 33] and dry eye syndrome [34, 35] have been published. Among these published studies, the application of ectoine to treat ophthalmic indications prompted us to perform a detailed analysis of the use of ectoine in this field. The ocular surface (cornea, conjunctivitis, and tear film) is a sensitive part of the human body exposed to various environmental challenges, such as heat, dry air, pollutants, or allergens. Besides these environmental exposures, individuals are sometimes genetically predisposed to—or develop—secondary inflammatory processes.

Allergic diseases, including allergic rhinoconjunctivitis, are a global health burden. The global prevalence of all allergic diseases is reported to be 20%-30% [36], resulting in a high pressure on the socioeconomic systems. The Global Allergy and Asthma European Network report indicated that cost savings of over EUR 100 billion could be realistically expected through better treatment of allergic diseases [37]. The 2008 and 2016 Allergic Rhinitis and its Impact on Asthma (ARIA) guideline supports physicians with a treatment algorithm for allergic rhinitis and conjunctivitis, depending on the severity and duration of the symptoms [38, 39]. Pharmacological therapies using oral/topical antihistamines, intranasal glucocorticosteroids (INGS), oral glucocorticosteroids, decongestants, and chromones are—beside avoidance—considered the keystones of allergic rhinitis and conjunctivitis treatment. Nevertheless, a relevant proportion of patients with symptoms are still not sufficiently treated [40–44]. A study reported that about 60% of allergic rhinitis sufferers in the U.S. are “very interested” in trying out new medications [45]. Furthermore, many patients are reluctant to use pharmacological therapies for fear of local irritations and side effects associated with sedative antihistamines, which in turn can lead to poor medication compliance [46, 47]. Therefore, nonpharmacological therapies with an advantageous tolerability and safety profile are of interest to many patients with allergic rhinoconjunctivitis.

Beside the allergic irritation of the eye surface, other noxious influences such as injuries, burn, or physical trauma also lead to inflammation and irritation of the air-facing epithelia, the conjunctiva, and cornea. Following the initial damage by noxae, inflammatory irritation leads to symptoms similar to the ones described for dry eye syndrome (DES). In most cases, DES symptoms develop as a consequence of a broad range of different causes and are not only limited to a preceding surgical intervention (cataract, strabismus correction, and laser in situ keratomileusis (LASIK) [48–52]), but DES can also occur in consequence of environmental influences, previous inflammatory diseases (chronic blepharitis, traumatic erosion of the cornea, keratitis of various etiologies, etc.), wearing contact lenses, and taking certain medications (anticholinergic drugs and antihistamines, alpha and beta blockers, antipsychotics, etc. [53]). In all mentioned cases, the initial disturbance of the eye surface is followed by a period of irritation and eye surface discomfort which contributes to a reduced quality of life. Together with the application of needed medication such as antibiotics (in case of surgery), steroids (in case of inflammation), or lubricants and wound healing promoting agents, the eye surface discomfort must be treated and a faster restoration period must be promoted.

Following the initial controlled trials on ectoine treatment of allergic rhinoconjunctivitis reviewed by Eichel [54] and first documented ectoine treatments of DES [35], several real-life, interventional, or noninterventional trials with ectoine-based eye drops have been conducted. In this article, we reviewed the literature regarding the treatment of irritations of the eye surface in the context of various indications. We focused on the ectoine treatment of allergic rhinoconjunctivitis and postsurgery treatment of ocular irritation and discomfort. The systemic review presented here is aimed at investigating the evidence on the use of this interesting substance for topical treatment of ocular surface irritations.

## 2. Methods

### 2.1. Objectives and Search Strategy

For this narrative review, the literature search was conducted in accordance with the Preferred Reporting Items for Systematic Reviews and Meta-Analysis (PRISMA) statement [55]. Primary databases were PubMed, Google Scholar, and Ovid. Initial search language was English. After the search in Google Scholar and PubMed that reported different articles on Ukraine language, we extended the search to Elibrary.ru and the National library of Ukrainian and Russian/Ukrainian language. The country of origin and languages were not limited. The time period was set to the beginning of 2010 until 22 July 2020.

**Figure 1:**
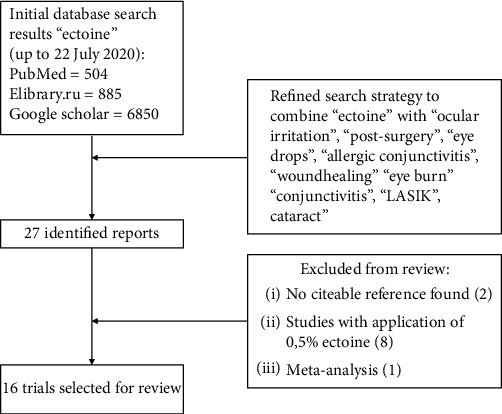
Flow chart of the literature selection process.

The following search terms/medical subject headings were: “ectoine” and “eye drops,” “ectoine” and “allergic conjunctivitis,” “ectoine” and “eye irritation,” “ectoine” and “allergic rhinoconjunctivitis,” “ectoine” and “eye burn,” “ectoine” and “wound healing,” “ectoine” and “eye,” “ectoine” and “LASIK,” “ectoine” and “Glaucoma,” “ectoine” and “cataract,” “ectoine” and “eye surgery,” and “ectoine” and “postoperative.” Studies published in peer-reviewed journals or presented on scientific congresses, reporting data on the role of the topical administration of ectoine eye drops to treat various irritations of the eye surface, were included. Studies related to other applications were not considered. Additional literature was found by reviewing the reference lists of the selected articles. The authors then independently assessed each publication and excluded those whose content was judged not to be strictly related to the subject of this review. They included only clinical trials where 2% ectoine eye drops were applied, controlled or uncontrolled, and interventional or noninterventional into eyes which were irritated by various reasons. Reviews, systematic reviews, meta-analyses, retrospective medical record reviews, case series, preclinical or observational studies, letters, editorials, technical notes, errata, and reports of pooled data were excluded (Figure 1).

### 2.2. Search Results

At the end of our selection process, 16 clinical studies [33, 56–70] were included, investigating the potential role of ectoine in allergic conjunctivitis, vernal conjunctivitis, functional epiphora, and irritation of the eye surface after external noxae/damage in a total of 1795 patients.

### 2.3. Study Design and Study Population

Except one trial [65] all were real-life studies investigating the application of 2% ectoine eye drops in different settings over a period of up to 6 months, either as monotherapy or in combination with other interventions.

Patient-reported outcome (diary) was used in all studies. The scores for patient reported outcome differed greatly in the method of reporting: from combined scores for all symptoms to individual scales for up to 8 symptoms. The summary scores were also calculated differently in the analysed studies. In addition to patient-reported outcomes, several trial and indication-specific parameters were measured and collected, such as wound closing time, reepithelisation time, or tear production (Schirmer test).

Regarding the study medication, an eye drop formulation with 2% ectoine, 0.35% hydroxyethyl cellulose, 0.35% NaCl, citrate buffer, and water was applied in all except one study. This study [71] used a formulation of 2% ectoine, 0.2% sodium hyaluronate, 0.35% NaCl, and water.

Used comparator products were systemic or local antihistamines [33, 68], standard of care (variety of drugs according to national guidelines), placebo [65, 72, 73], and fluorometholone [61].

The overall study design differed greatly between studies. Most of the studies did not comment on randomization (9/16). The number of study arms ranged from single-arm trials (*n* = 4) [56, 59, 62, 63, 74] over 2 armed trials (*n* = 10) to one study with 4 arms [70]. One study used historic controls [61].

A total of 1795 subjects were studied in the 16 different trials. Of those, 1225 applied ectoine eye drops during their respective observation period. Four clinical trials specifically studied the effect of ectoine in children and adolescents (524 subjects in total with 492 using ectoine eye drops); the youngest child included was 2 years of age. Two other trials included children and adults but did not publish the age of the participants. Fourteen of the 16 studies included both, males and females. Two studies included only male patients. Most of the studies were performed in the Ukraine but also in Germany, Poland, Canada, Spain, and Italy. Details on the studies can be found in Tables 1 and 2.

## 3. Results from Included Clinical Trials

### 3.1. Safety of Ectoine Eye Drops

All 16 studies evaluated the safety of ectoine eye drops. This is of particular interest since very sensitive patient groups, like children from the age of 2 years and patients with a very recent eye surgery, were investigated in some studies. In detail, 4 studies especially treated children and adolescents with ectoine eye drops, and 2 studies evaluated the eye drops in children, adolescents, and adults. None of the studies reported a serious adverse event (SAE). Only a small number of adverse effects/adverse events (AEs) were reported. All authors attributed an excellent safety profile towards the ectoine eye drops (Tables 1 and 2).

### 3.2. Efficacy of Ectoine Eye Drops (EED)

The 16 studies reviewed can be divided into two main groups:

- Studies investigating treatment of allergic conjunctivitis with EED [33, 56, 57, 59, 62, 65, 68] (Table 1)
- Studies investigating treatment of nonspecific irritation of the eye surface with EED [58, 60, 61, 63, 64, 66, 67, 69, 70] (Table 2)

## 4. Discussion

### 4.1. Study Design and Available Information

This review showed that ectoine eye drops (EED) have been successfully applied in a range of clinical studies covering different indications. Many of these studies are not yet internationally published (especially the Ukrainian studies), and detailed information on some of the studies (8 out of 16) was not available, as only presentations from scientific congresses could be obtained and reviewed. Therefore, an in-depth analysis of the presented data was not possible for these studies. For most of the studies, information regarding randomization or blinding is missing, nor do entries in international study databases exist for 14 of the 16 studies. However, the combined data from fully published studies together with the data from conference presentations allowed a review of the application of EED in nonspecific irritation or inflammation of the eye surface including allergic conjunctivitis.

All 16 studies reviewed here applied an eye drop formulation with 2% ectoine as key ingredient. As 15 studies applied the same formulation and the remaining one differed mainly in the type of viscosity enhancer in the formulation, this study could still be included as a confirmatory study, demonstrating that the efficacy of 2% ectoine is not dependent on the lubricant used in the formulation.

We found two main areas of ocular irritation and inflammation where EED have been studied: (1) allergic conjunctivitis and (2) nonspecific eye irritation and inflammation caused by physical damage to the eye (e.g., surgery or burn).

Within the scope of allergic conjunctivitis, 5 studies investigated the effect of EED in seasonal allergic rhinitis [33, 59, 62, 65, 68], and 2 studies included patients with vernal conjunctivitis [56, 57]. Primary outcome parameters of all studies were patient-reported symptoms together with study-specific measurements related to the respective study endpoints. The studies differed in terms of the studied populations (children, adults), EED application period (1 week up to 6 months), and design (comparative, crossover, add-on, noncomparative, with parallel treatments of eyes and nose, retrospective case series) with only one trial being placebo controlled. The overall number of participants for the 7 studies analysing allergic conjunctivitis was 444, including 254 children. Interestingly, one study enrolled 192 patients whereas the other 6 trials included 42 patients on average.

All studies investigating allergic conjunctivitis showed a significant improvement of the patient-reported outcome following application of EED, which was significantly better than placebo [65] and at least comparable to pharmacological standard treatments such as ketotifen or azelastine [33, 68]. Usage together with standard therapy resulted in a faster decrease of symptoms than standard therapy alone (e.g., reduction of itching in 2.2 days versus 4.0 days, complete resolution in 5.3 days versus 12.8 days) [68]. Interestingly, one long-term application in patients with vernal conjunctivitis delayed the use of corticosteroids in 75% of the patients [56]. All 7 studies reported a very good tolerability and safety of the EED, both in children and in adults, which was even significantly better than an established over-the-counter drug such as ketotifen or azelastine [33, 57].

As shown above, the overall picture described by the reviewed studies is a good efficacy of EED in treating the symptoms of allergic conjunctivitis either as monotherapy or in combination with other interventions, together with a very good tolerability and excellent safety profile. Especially, the results of an add-on effect of ectoine when used together with pharmacotherapies are of interest, as combination therapies are suggested by different studies in allergic rhinitis. The revision of the ARIA guideline in 2016 recommends the combination of intranasal/oral antihistamine and INGS; the combination of INGS and intranasal antihistamines acts faster than INGS alone and thus might be preferred by patients [39]. The combination of oxymetazoline and mometasone furoate nasal spray showed greater reductions in allergic rhinitis symptoms than mometasone furoate nasal spray alone [75]. Greiwe and Bernstein [76] concluded that two combinations—intranasal antihistamine with INGS and INGS with nasal decongestants—are advantageous for patients with complex rhinitis symptoms in terms of symptom control. Similar results are to be expected for a combination treatment of allergic rhinoconjunctivitis.

The remaining 9 studies investigated nonspecific eye irritation and inflammation of the ocular surface after a harming impact such as surgery, eye burn, or unclassified disturbance. In 7 studies, treatment with EED was conducted in the postoperative phase for different reasons (strabismus, traumatic injuries, and advanced keratoconus) [58, 60, 63, 64, 67]. One study investigated the effects on functional epiphora of unknown origin [61], another on healing after eye burn [66] and one study on the effects during an aseptic uveitis after penetrating injury [67]. A subgroup analysis on irritation due to long-term use of contact lenses was also done in one study [70]. Again, with a total of 268 children and adolescents in 2 studies exclusively conducted in this population [64, 67], the EED was applied to a very sensitive group of patients.

One hallmark result from all studies was the positive effect of EED on wound healing and reepithelization in the respective studies: Pastukh et al. [63], Gorokhovskaya et al. [60], and Sarzhevska and Tabakovа [66] reported a faster healing when EED was applied concomitantly to the conventional regime after eye damage, and Rykov et al. reported positive effects on postoperative scar reduction [64]. All 8 studies conducted during the post damage or post-operative phase reported positive effects of the EED compared with standard treatment only or even to sodium hyaluronate instillation [66]. These irritation/inflammation symptoms of the ocular epithelium like conjunctival hyperaemia or foreign body sensation are often referred to as secondary dry eye syndrome. These results are supported by the study of Martinez et al., in which a comparable efficacy of EED with fluorometholone in treating functional epiphora symptoms was shown without having the typical negative side effects like interocular pressure, which is associated with corticosteroid treatment [61].

### 4.2. Mode of Action of Ectoine as Ideal Qualification for Its Ophthalmic Application

**Figure 2:**
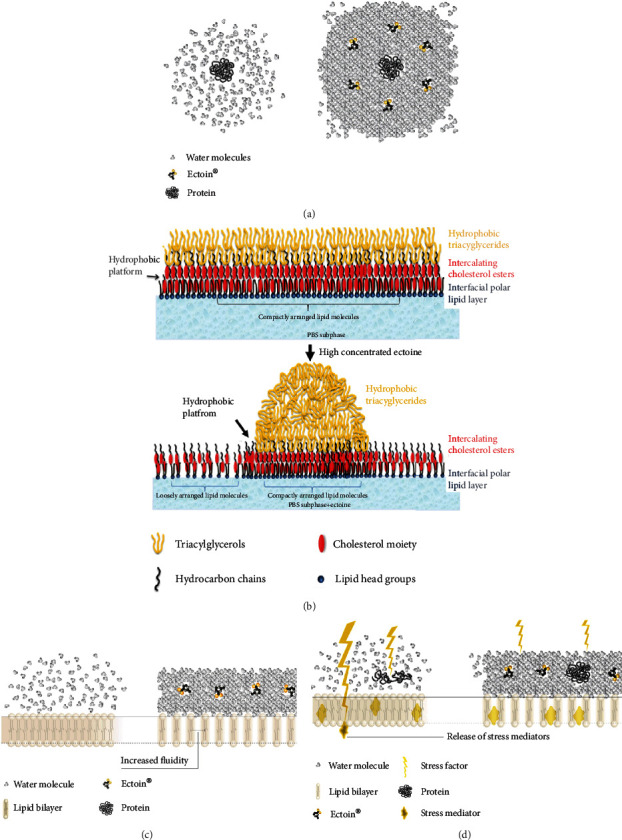
Ectoine's mechanism of action. (a) Influence on water molecules and proteins: in the presence of ectoine, the water structure is altered and more compact, and proteins are stabilised. (b) Molecular model explaining the effect of ectoine on the tear fluid lipid layer (picture taken from Dwivedi et al.) [19]. (c) Model of effects of ectoine on cell membranes (lipid bilayers) without external stress. (d) Protective effect of ectoine against external damage (e.g., allergens, UV-light, and physical damage): the ectoine water interaction results in the protection of cell membranes (lipid bilayers), thus leading to reduced release of stress mediators.

The results from the clinical trials presented here are in line with the mode of the action model of ectoine, which was reported by different researchers. Based on the “preferential exclusion” model presented by Arakawa and colleagues [5] and reviewed by Lentzen and Schwarz [2], ectoine exerts its protective function by its cosmotropic effect on water molecules and—when applied topically to epithelia—results in the stabilisation of the respective tissue (Figure 2).

This stabilisation results in a reduction of inflammation, as seen in the reduction of particulate matter-induced inflammation of lung epithelia [15] and in UV-induced inflammation of the skin [77]. The effective treatment of upper respiratory tract infections has recently been reviewed [23], and effects on inflammatory diseases of the lung were also published [26, 29]. Applied on the ocular surface, ectoine stabilizes not only the tissue but also the meibum layer as presented recently [18–20, 22]. This effect, both on epithelium and on the surrounding tear film, may explain the broad and unspecific positive effect of ectoine on irritations and inflammation of the ocular surface. Moreover, it allows an add-on treatment to pharmacological treatments, thus making use of different pathways, resulting in additional effects.

Interestingly, different studies were identified during this review, which reported on positive outcomes of application on ectoine-based formulations on dry eye syndrome [34, 35]. Although these trials are not subject of this work and were excluded, it is worth to mention that additional evidence is available proving the successful treatment of the ocular surface with ectoine.

## 5. Conclusions

In this review, we provide evidence based on the review of 16 independent studies from 6 countries that irritations and inflammations of the ocular surface can be treated with ectoine-based eye drops, either alone or in combination with other (pharmacological) therapies. Although many of the studies showed limitations regarding their study design or reporting and data is not fully available, the following readout can be supported: ectoine is a natural substance with a unique mode of action on the eye surface and with an excellent tolerability and safety profile. This conclusion is supported by the work of other colleagues, who reviewed the efficacy of ectoine in upper respiratory inflammation [23] or allergic rhinitis (systematic review submitted) and other indications. Especially, the studies on dry eye syndrome, which have not been subject of this review, should be analysed in detail to further strengthen the evidence base for ocular application of ectoine.

This systematic review of the literature extends the previously existing knowledge about the substance in two ways.

On the one hand, some of the articles cited in the review report on patients with allergic conjunctivitis or rhinoconjunctivitis treated in real-world scenarios. This means that many of the patients had concomitant diseases and were taking concomitant medications for the underlying pathology or for other coexisting diseases. From this real-world evidence, we can conclude that the effects demonstrated in these situations are generally the same as those observed before in the more selected populations of the controlled trials.

On the other hand, these recently published articles extend our knowledge into the area of new indications beyond allergic conjunctivitis. There are reports about the use of ectoine in traumatic uveitis following a penetrating injury of the eye and similar traumatic situations. Also, the substance was studied in the vulnerable postoperative state of the eye following surgical interventions of the cornea or in corrective strabismus operations, as well as postburn treatment. From these observations we learn that, in the surgical field of ophthalmology, traumatology, nonsurgical damages, or irritations of the eye surface, the very special properties of ectoine regarding the restoration of barrier functions may open a new perspective for this treatment modality.

Therefore, ectoine-based eye drops represent a viable alternative or add-on treatment option for nonspecific eye irritation and ocular inflammation acting through stabilisation of the epithelial barrier of the organ.

## Figures and Tables

**Table 1:** Trials studying the treatment of allergic conjunctivitis with EED.

Authors, year, country, and type of publication	Indication	Study design	Patient distribution & treatment	Study population Age range Mean age	Description of therapy, duration, and dosage	Efficacy parameters	Main findings (ocular symptoms)	Side effects
Salapatek et al., 2011 [65], Canada, Conference presentation (manuscript accepted)	Allergic rhinoconjunctivitis	Randomized, double-blind, placebo-controlled, double crossover	46 patients	Adults Age range: 22-65 years Mean age: n.a.	14 days of treatment with either EED/Ectoine Nasal Spray (ENS) or control (3 times per day), wash-out (1 week without treatment), crossover of the groups, 14 days treatment (3 times per day)	Patient reported outcome: Sneezing, nasal congestion, itchy nose, runny nose, watery eye, itchy eye, red eye, itchy ear/palate Total Nasal Symptom Score (TNSS), Total Ocular Symptom Score (TOSS), Total Nonnasal Symptom Score (TNNSS)	Patients receiving ectoine treatment experienced a greater relief of overall ocular symptoms scores during posttreatment experimental exposure chamber (EEC) when compared to placebo. The TOSS significantly decreased to 12.64 ± 0.97 (-24.4%; *p* = 0.0001) in the ectoine group and to 14.09 ± 0.91 (-15.8%) in the placebo group. Individual ocular symptoms were more reduced after ectoine treatment than with placebo, with a greater relief for “watery eyes” (*p* = 0.020) and “itchy eyes” (*p* = 0.021)	6 AEs reported during EED/ENS treatment. During placebo treatment 5 AEs were reported. No SAE occurred.
Werkhäuser et al., 2014 [33], Germany, Peer-reviewed publication	Allergic rhinoconjunctivitis	Controlled, noninterventional, open-labelled, multicentre	48 patients Ectoine group: 22 Azelastine group: 26	Adults Age range: n.a. Mean age: 35 years	7 days of treatment with either EED: 1 eye drop per eye and 1 puff of the nasal spray per nostril 4 times per day, or azelastine (0.05 to 0.1 mg/L): 1 eye drop, 1 puff nasal spray, both twice per day	Investigator and patient assessment of nasal obstruction, rhinorrhoea, sneezing, nasal itching, conjunctivitis, eye itching, tearing, alate itching, TOSS, TNSS, efficacy judgement, tolerability judgement	The TOSS in the investigator assessment decreased significantly from V1 to V2 in both groups (*p* < 0.001 for EED, *p* = 0.009 for azelastine). TOSS values decreased in the ectoine group by 45.96% and by 44.98% in the azelastine group. Decreases of TOSS values as assessed by patients were not significant	8 AEs in total, 2 in the ectoine group and 6 occurred in the azelastine group. No SAE occurred
Mrukwa-Kominek et al., 2018 [62], Poland, Conference presentation	Allergic conjunctivitis	Single-arm, open-labelled, noninterventional	30 patients Ectoine group (30)	Adults Age range: 21-75 years Mean age: 44.8 years	14-21 days of treatment One eye drop per eye up to 4 times per day	Assessment included McMonnies questionnaire, evaluation of therapeutic efficiency and adverse effects, best corrected visual acuity, intra ocular pressure, slit lamp examination with fluorescein eye stain test, ocular surface disease index, vision related quality of life	Treatment with ectoine led to significant improvement for conjunctival redness, a reduction of follicular reaction and reduction of eyelid oedema, and a significant decrease of individual ocular symptoms. McMonnies questionnaire showed a 15% reduction of symptom score	Treatment tolerance in patients with allergic conjunctivitis was good with very few adverse effects
Allegri et al., 2014 [57], Italy, Conference presentation	Vernal keratoconjunctivitis (VKC)	Retrospective case series, controlled	64 patients Ectoine group (32) Ketotifen group (32)	Male children Age range: n.a. Mean age: 8.5 years	6 months of treatment Ectoine: 1 eye drop per eye, 3 times per day Ketotifen (0.05%) 1 eye drop per eye, 3 times per day	Assessment included VKC slit-lamp signs: Focal or diffuse conjunctival hyperaemia, tear break up time, modified Oxford scale, VKC grading (modified Bonferroni scale) and symptoms: VAS scale grading (ocular pain, itching, tearing, photophobia and foreign body sensation), quick questionnaire on tolerance of eye drops at instilment	The case series review showed that both treatments (2% ectoine and 0.05% ketotifen) are effective in improving signs and symptoms of VKC during allergic seasons. In tolerability rating, ectoine was significantly better rated (*p* < 0.0001)	None reported
Drozhzhyna and Troychenko, 2015 [59], Ukraine, Publication	Allergic conjunctivitis	Real-life, uncontrolled, noninterventional	30 patients Ectoine group (30)	Adults Age range: 18-65 years Mean age: n.a.	7-14 days of treatment as prescribed (one eye drop per eye up to 4 times per day)	Assessment included Symptoms of conjunctival hyperaemia, lacrimation, and ocular itching. Conjunctival hyperaemia and oedema were evaluated by the ophthalmologists, whereas lacrimation and ocular itching were documented by the patients	After treatment, the scores for conjunctival hyperaemia, ocular itching, eyelid oedema, and lacrimation decreased significantly (*p* < 0.05). Eyelid oedema was significantly improved in all 30 patients (*p* = 0.01) and completely resolved in 22 patients at the end of the study	All patients experienced good tolerance to ectoine eye drops, with no side effects being reported
Skrypnyk and Seidametova, 2017 [68], Ukraine, Publication	Allergic conjunctivitis	Randomized, controlled	34 patients Ectoine + standard of care (24) Standard of care (10)	Adolescents and adults Age range: 13-42 years Mean age: n.a.	Ectoine group: 2 weeks before onset of symptoms and during exacerbation as prescribed control group: traditional treatment from the moment of exacerbation	Symptoms were assessed on a 4-point scale: 0 = no symptoms, 1 = mild symptoms, 2 = moderate symptoms, 3 = severe symptoms	In the ectoine group, the symptoms of ocular itching, conjunctival hyperaemia, and oedema improved significantly faster compared the control group (*p* < 0.05)	No AE reported, a good tolerance of the eye drops was reported
Allegri et al., 2018 [56], Italy, Conference presentation	Vernal keratoconjunctivitis	Retrospective	Ectoine: 192	Children Age range: up to 10 years Mean age: 7.8 years	6 months of treatment time as prescribed (1 eye drop per eye, 3 times per day)	Assessment of the preventive administration of ectoine eye drops to shorten the duration of VKC relapses or to mitigate the attacks	8% of the included subjects had no relapse of VKC, 38% needed topical corticosteroid or cyclosporin treatment, but it was started 2 months later compared to previous years, 29% needed these topical drugs 3 months later, and 25% had a similar to previous year course	The treatment was well tolerated, and only 1 child had to stop it because of a local reaction to the eye drops

n.a.: not available.

**Table 2:** Studies applying EED in nonspecific irritation or inflammation of the eye surface.

Authors, year, country, and type of publication	Indication	Study design	Patient distribution & treatment	Study population Age range Mean age	Description of therapy, duration, and dosage	Efficacy parameters	Main findings (ocular symptoms)	Side effects
Serdyuk et al., 2017 [67], Ukraine, Publication	Aseptic uveitis following a penetrating injury	Prospective, controlled	24 patients Ectoine + standard of care group (14) Standard of care group (10)	Children & adolescents Mean age: 10.6 years	1-week treatment time Ectoine: 8 times per day Control: as prescribed	Assessment of visual acuity, biomicroscopy of the front part of the eye and optical media, daily ophthalmoscopy, ultraviolet examination and plain radiography of the eye sockets, conjunctival redness, and photophobia based on a score	On the 8th day of treatment, an improvement in the condition of all patients in both groups was observed, and the severity of the inflammatory response decreased in both groups (*p* < 0.01). IL-1 and CRP levels decreased in both groups, but significantly stronger in the ectoine group (*p* < 0.05)	No side effects were observed from the use of ectoine
Ustimenko et al., 2017 [69], Ukraine, Conference presentation	Early postoperative period in patients with advanced keratoconus	Prospective, comparator controlled	24 patients Ectoine (10) Sodium hyaluronate (10)	Adults (male) Age range: n.a. Mean age: 23 years	1-month treatment time Ectoine: 3 times per day Sodium hyaluronate: 3 times day	Corneal epithelialization was determined by optical coherence tomography; the severity of the corneal syndrome by a subjective score	The corneal syndrome severity in the ectoine group was 2.2 points lower than in the control group after 3 and 5 days on average and 0.5 points after 7 days. After a month, in the first group the cornea was transparent (0 points) in 100% of patients, in the second group -1 point in 70% of patients, and in 30% -2 points	No AE reported
Sarzhevska and Tabakova, 2017 [66], Ukraine, Conference presentation	Eyeball burns of different origin	Prospective, controlled	49 patients Ectoine + standard of treatment (24) standard of treatment (25)	Adults Age range: 18-57 years Mean age: n.a.	14 days of treatment time Ectoine: 3-4 times per day Control: as prescribed for standard of care	The clinical effect was evaluated by the duration of objective and subjective improvement, time before epithelialization, and intensity of the corneal opacity; nature and number of complications; and eye function improvement	The ectoine eye drop-combined therapy resulted in shortened epithelialization rates by 3-4 days. Ectoine use allowed to eliminate inflammation 4.2 days earlier (*р* < 0.05). Visual acuity of patients from the ectoine group was 23.2% higher than that of patients from the control group. Analysis of the late effects showed that the corneal opacity was 22.9% less common in the ectoine group than in the control group	No AE reported
Rykov et al., 2018 [64], Ukraine, Publication	The course of the inflammatory reaction and cosmetic outcomes of the postoperative period in children who received strabismus surgery	Prospective, comparator controlled, single crossover	234 patients/264 eyes	Children & adolescents Age range: 2-18 years Mean age: n.a.	3 months of treatment time Ectoine: 3 times per day Control: as prescribed	The severity of the inflammatory reaction was determined via subjective scoring in 4 categories: hyperaemia, oedema, lacrimation and discharge. The cosmetic effect was evaluated 1 month and 3 months after the surgery by scoring of the scar	After similar inflammatory status after surgery between both groups, lacrimation, discharge, and overall score were significantly better in the ectoine group after 21 days (*p* ≤ 0.03) with almost no signs of inflammation. The postoperative scar (cosmetic effect) was almost imperceptible in the ectoine group after one month, while in the control group a clearly noticeable scar was observed on the conjunctiva after 1 month	No AE reported
Vitovskaya et al., 2018 [70], Ukraine, Publication	Treatment of traumatic injuries of the eye surface and secondary dry eye syndrome due to contact lens wear	Prospective, 4 groups, comparator controlled	100 patients Group 1: ectoine + Additional therapy: antiseptics, antibiotics (25) Group 2: ectoine Complementary therapy: antiseptics, anti-inflammatory and anti-infective drugs, wound healing promoting agents (25) Group 4: ectoine (25) Group 4: no treatment (25)	Adults Age range: 18 -40 Mean age: 21	Treatment time was 3 months Group 1: ectoine 4 times per day Group 2: ectoine 5 times per day Group 3: ectoine 3 times per day Group 4: no treatment, healthy	Assessment included Patient complaints, biomicroscopic studies, diagnostic tests	Ectoine eye drops provide normalization of the precorneal tear film and increase tear production in people with traumatic eye surface pathology	None reported
Bondarenko, 2018 [58], Ukraine, Conference presentation	Effectiveness of treatment after phacoemulsification of cataract (FEC) compared with baseline anti-inflammatory therapy	Prospective, comparator controlled	786 patients Ectoine group (374) Control group (412)	Adults Age range: n.a. Mean age: n.a.	Treatment time was 5 weeks of instillations of ectoine eye drops with 2%, 1 drop 6 times a day during the first week and from the second week -5 times a day with 0.5% ectoine; and 4, 3 and 2 times a day with one drop of 0.5% ectoine for the third, fourth and fifth weeks, respectively	Evaluation criteria were conjunctival hyperaemia, corneal condition, and subjective evaluation of patients (sensation of foreign body, dryness, and discomfort) on the second day and one month after the FEC	2 days after FEC, hyperaemia was absent in 256 eyes (62.13%) of the control group and 338 eyes of the ectoine group (90.3%). Corneal state was transparent with 286 (76.4%) eyes in the ectoine and 248 (60.1%) eyes in the control group. After 1 month, 43.4% of the patients in the control group reported discomfort, compared to 0% in the ectoine group	None reported
Gorokhovskaya et al., 2018 [60], Ukraine, Conference presentation	Treatment of posttraumatic corneal erosion	Prospective, controlled	80 patients Ectoine (50) Control (30)	Adults Age range: 24-70 years Mean age: n.a.	Treatment time 14 days Ectoine 4 times per day Control/standard of care as prescribed	A daily examination of visual acuity, biomicroscopy (area of deepithelization of the damaged cornea), fluorescein staining, examination of the fundus	The complete epithelization of the defect of the damaged cornea occurred on average after 5.2 days in the control group and 3 days in the ectoine group with less complaints on discomfort, pain, lacrimation, photophobia, and a foreign body sensation in the ectoine group	None reported
Pastukh et al., 2019 [63], Ukraine, Conference presentation	Rehabilitation after eye surgery	Prospective, open-label	32 patients Ectoine: 32	Children & adults Age range: 8 to 69 years Mean age: n.a.	30 days of treatment ectoine: 3 times per day	Assessment included Corneal epithelization process, presence of corneal oedema, severity of pain, visual acuity	Foreign body sensation, itching or burning, and moderate swelling of the cornea in the area of postoperative wounds were observed for 2-3 days; they significantly decreased after instillation of ectoine eye drops. Complete epithelization of the surface layers of the cornea was observed within 7 days	None reported
Martinez et al., 2019 [61], Spain, Publication	Functional epiphora	Prospective, historic controlled, observational trial	26 patients Ectoine: 26 Fluorometholone (FML): 26 (historic control)	Adults Age range: n.a. Mean age: 64 years	1 month of treatment time Ectoine: 3 times per day Fluorometholone as prescribed	Measurement of functional epiphora reduction after treatment	The “no inferiority” study shows that treatment of functional epiphora with ectoine eye drops provides efficacy and is as effective as corticosteroid eye drops (fluorometholone)	No side effects occurred; measurement of intraocular pressure showed no negative effect

## Data Availability

Data sharing is not applicable to this article as no datasets were generated or analysed during the current study.

## Abbreviations

AE: Adverse event
ARIA: Allergic rhinitis and its impact on asthma
CRP: C-reactive protein
DES: Dry eye syndrome
EEC: Environmental exposure chamber
EED: Ectoine eye drops
ENS: Ectoine nasal spray
FEC: Phacoemulsification of cataract
FML: Fluorometholone
INGS: Intranasal glucocorticosteroids
LASIK: Laser in situ keratomileusis
PRISMA: Preferred Reporting Items for Systematic Review and Meta-Analysis
OTC: Over-the-counter
SAE: Serious adverse event
TNSS: Total Nasal Symptom Score
TOSS: Total Ocular Symptom Score
VKC: Vernal Keratoconjunctivitis.
